# Neural mechanisms of guided internet-based cognitive behavioral therapy for children with attention-deficit/hyperactivity disorder: a protocol for a prospective multimodal MRI study

**DOI:** 10.3389/fpsyt.2026.1917984

**Published:** 2026-09-23

**Authors:** Junbing He, Sayo Hamatani, Qiulu Shou, Yoshifumi Mizuno

**Affiliations:** 1 Research Center for Child Mental Development, University of Fukui, Fukui, Japan; 2 United Graduate School of Child Development, The University of Osaka, Osaka, Japan; 3 Division of Developmental Higher Brain Functions, United Graduate School of Child Development, University of Fukui, Fukui, Japan; 4 Department of Child and Adolescent Psychological Medicine, University of Fukui Hospital, Fukui, Japan

**Keywords:** attention-deficit/hyperactivity disorder, children, internet-based cognitive behavioral therapy, multimodal magnetic resonance imaging, study protocol

## Abstract

**Introduction:**

Attention-deficit/hyperactivity disorder (ADHD), a common neurodevelopmental disorder in childhood, is associated with academic, social, and emotional difficulties. Although pharmacotherapy is widely used for treating ADHD, functional impairments often remain. Cognitive behavioral therapy (CBT) has demonstrated benefits in children with ADHD, and internet-based CBT (ICBT) may improve treatment accessibility. However, the neurobiological mechanisms underlying ICBT remain unclear, and neural markers predicting treatment responses have not been well established, particularly in children with ADHD. Neuroimaging may aid in the exploration of changes in the brain structure and function that are associated with interventions and provide insights into predictors of treatment response.

**Methods and analysis:**

This multimodal neuroimaging study aims to examine intervention-related neural changes in children with ADHD using pre- and post-intervention MRI assessments. Participants will be drawn from a cohort of participants of an ongoing randomized, sham-controlled ICBT clinical trial and will thus belong to an ICBT or sham control group. Approximately 60 children with ADHD will undergo multimodal magnetic resonance imaging (MRI), including structural MRI, resting-state functional MRI, and diffusion tensor imaging before and after an 11-week intervention. MRI data from typically developing (TD) children will be obtained from an ongoing neuroimaging database for baseline cross-sectional comparisons and as a reference for interpreting post-intervention neural changes in the ADHD group. Analyses will explore intervention-related changes in brain structure and function, as well as associations between baseline MRI markers and clinical treatment response.

**Ethics and dissemination:**

This study was approved by the Research Ethics Committee of the University of Fukui Hospital in February 2026 (ethics approval number: 20250238). Participant recruitment commenced in March 2026. The study findings will be disseminated through peer-reviewed journals and scientific conferences.

## Introduction

Attention-deficit/hyperactivity disorder (ADHD) is a common neurodevelopmental disorder in childhood characterized by persistent symptoms of inattention, hyperactivity, and impulsivity. Global prevalence estimates indicate that ADHD affects approximately 7.2–7.8% of children and adolescents, with a prevalence of 11.7% among children aged 3–17 years in the United States (1, 2). These ADHD symptoms frequently interfere with academic performance, peer relationships, and family functioning and often persist into adolescence and adulthood (3). Children with ADHD are at increased risk of emotional dysregulation, comorbid anxiety, depression, and reduced quality of life (2, 4). Therefore, early ADHD detection and intervention are essential.

Pharmacotherapy is widely used as part of treatment for children with ADHD (5). However, ADHD symptoms often persist despite medication (6, 7), and evidence for the long-term effectiveness of currently available pharmacotherapy remains limited (6, 8). Thus, psychosocial interventions are considered important components of comprehensive treatment approaches for children with ADHD. Cognitive behavioral therapy (CBT) has demonstrated clinical efficacy in children with ADHD, although treatment responses vary across individuals and studies (9, 10). Because access to face-to-face CBT for children with ADHD is often limited, internet-based CBT (ICBT) has been developed to address this limitation (11). In Japan, ICBT has demonstrated effectiveness across several psychiatric conditions (12, 13). Given the widespread use of digital devices among school-age children in Japan, ICBT may be particularly suitable for children with ADHD.

Despite growing clinical interest, the neurobiological mechanisms that underlie ICBT-induced behavioral changes in children with ADHD remain poorly understood. Neuroimaging studies using magnetic resonance imaging (MRI) have shown that, compared with healthy controls, patients with ADHD exhibit structural and functional brain alterations (14). Structural MRI studies have reported changes in gray matter volume (GMV) in the frontal, temporal, parietal, and subcortical regions (15, 16). Resting-state functional MRI (rs-fMRI) studies have further indicated altered functional connectivity (FC) within and between large-scale networks such as the default mode network and those related to attention, cognitive control, and reward processing (17, 18). In addition, diffusion MRI studies have suggested the involvement of atypical white matter microstructures in the frontostriatal tracts, corpus callosum, and other major tracts related to attention and executive control in ADHD, highlighting the role of distributed brain networks in ADHD (19). Collectively, cross-sectional neuroimaging studies have consistently reported structural and functional brain alterations in ADHD, although the findings remain heterogeneous. However, cross-sectional designs are limited in their ability to account for potential confounding factors and do not allow for inference regarding temporal or causal relationships. In contrast, longitudinal and intervention-based studies provide an opportunity to examine temporal changes and are better suited to investigating potential causal mechanisms. Therefore, longitudinal neuroimaging studies are needed to further elucidate brain changes associated with development and treatment.

Longitudinal neuroimaging studies have further demonstrated that certain neural measures may change during development or in response to pharmacological treatment. For example, a higher cumulative methylphenidate dosage has been associated with GMV increases in the frontal regions implicated in executive control (20), and methylphenidate treatment has been reported to increase spontaneous neural activity in the nucleus accumbens and within large-scale functional networks, including the salience and default mode networks (21). As noted above, most longitudinal neuroimaging studies on ADHD have focused on pharmacological treatments. In contrast, longitudinal neuroimaging studies examining the neural changes following cognitive-behavioral or internet-based interventions remain limited, particularly in children with ADHD. To the best of our knowledge, no longitudinal neuroimaging study has examined post-CBT neural changes in children with ADHD. Given the lack of pediatric evidence, neuroimaging studies examining post-intervention brain changes in children with ADHD are warranted. Against this background, examination of pre- and post-intervention MRI data from children with ADHD offers a unique opportunity to investigate the neural changes associated with psychological intervention.

In addition to characterizing intervention-related neural changes, identifying the predictors of treatment response is an additional clinical priority. Although neuroimaging features have been explored as potential predictors, treatment response varies substantially across individuals, and reliable neurobiological predictors have not yet been established, particularly in ADHD (22–24). In ADHD, emerging evidence from pharmacological studies suggests that baseline structural and diffusion MRI measures may be related to variability in stimulant treatment responses, including findings from conventional imaging models and radiomics-based approaches (25). Despite these advances, longitudinal neuroimaging research investigating the baseline predictors of responses to non-pharmacological interventions remains limited, particularly in children with ADHD. Therefore, it remains unclear whether similar predictive frameworks can be extended to psychological or internet-based treatments in this population.

Thus, building on prior research and existing limitations in the literature, this study is designed as a nested, longitudinal neuroimaging study conducted in parallel with an ongoing randomized, sham-controlled clinical trial evaluating the clinical efficacy of ICBT in children with ADHD. The findings of this study are expected to improve our understanding of the neural mechanisms underlying ICBT, contribute to the identification of neuroimaging biomarkers associated with treatment response, and support future precision psychiatry and individualized treatment strategies for children with ADHD.

### Aims and hypotheses

The primary aim of this study is to examine intervention-related changes in brain structure and function, assessed by multimodal MRI, following an ICBT program in children with ADHD and compare these changes with those after a sham control program. In addition, this study aims to characterize the baseline differences between the brains of children with ADHD and typically developing (TD) children, and to examine post-intervention neural patterns in the ICBT group relative to this reference. Furthermore, this study will explore baseline neuroimaging markers as potential predictors of treatment response in children receiving ICBT.

We hypothesize that ICBT will be associated with intervention-related changes in brain structure and function relative to the sham control condition, and that baseline neuroimaging features will be associated with symptom improvement following ICBT and may serve as potential predictors of treatment response. Specific patterns and directions of these effects are not prespecified.

## Methods and analysis

### Study design

This study describes a multimodal neuroimaging protocol designed to assess measures of brain structure and function obtained before and after an intervention. The participants of this neuroimaging study will be a subset of individuals randomized in an ongoing sham-controlled ICBT clinical trial with a parallel-group design. MRI data, including T1-weighted (T1w) imaging, rs-fMRI, and diffusion tensor imaging (DTI), will be acquired before and after the intervention period. The flow diagram of the study is shown in **Figure 1**. An overview of all assessments included in this study, together with their planned schedules, is provided in **Table 1**. A TD reference group will be included using data from an ongoing neuroimaging database to enable baseline cross-sectional and reference-based post-intervention comparisons.

**Figure 1:**
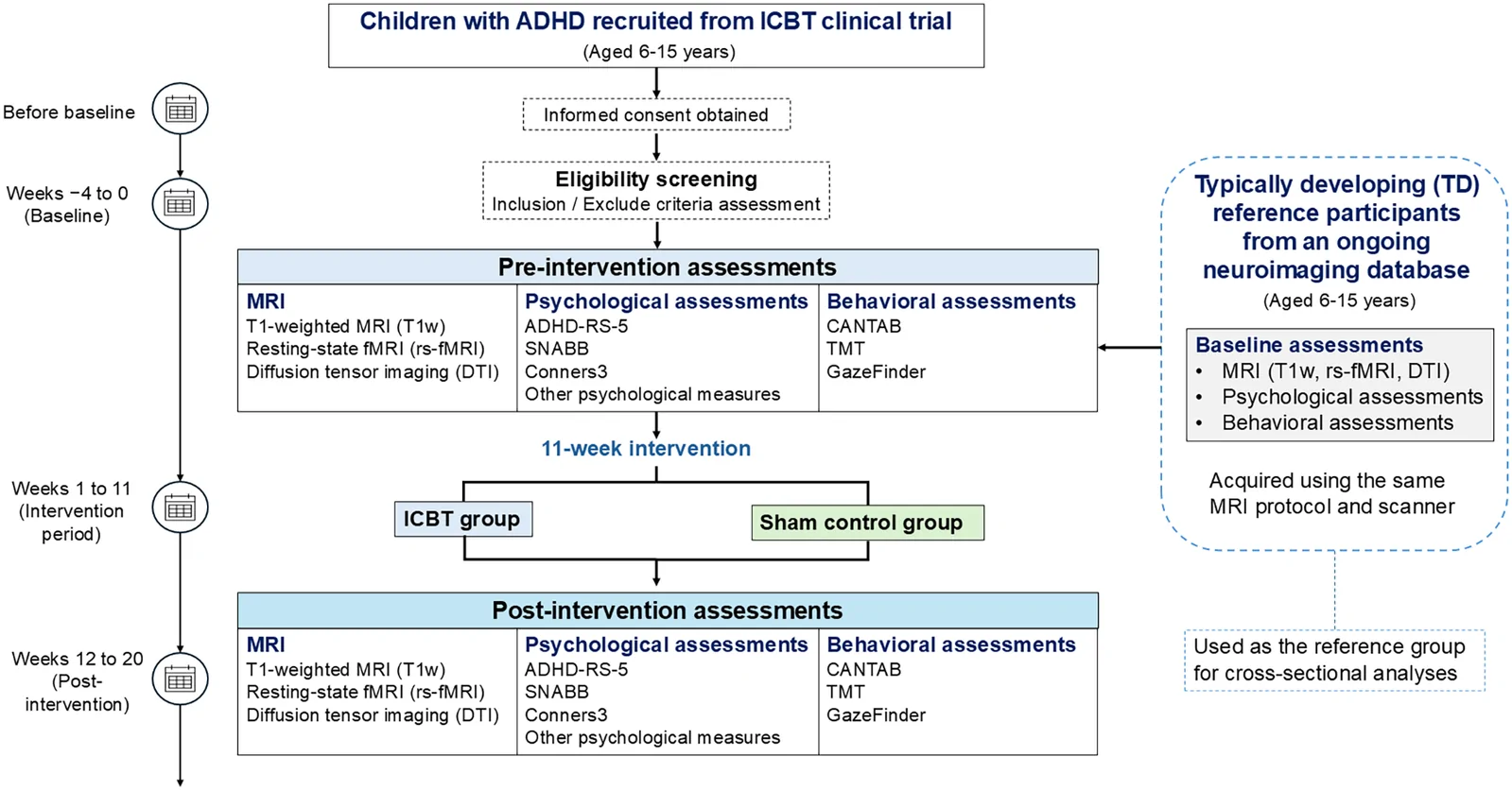
Study design and longitudinal MRI assessment protocol for children with ADHD. ADHD, attention-deficit/hyperactivity disorder; DTI, diffusion tensor imaging; ICBT, internet-based cognitive behavioral therapy; MRI, magnetic resonance imaging; rs-fMRI, resting-state functional MRI; T1w, T1-weighted; TD, typically developing.

**Table 1:** Standard protocol items.

Item	Enrollment	Pre-intervention (baseline)	Intervention	Post-intervention
Time point	Before baseline	−4 to 0 W	1 to 11 W	12 to 20 W
Informed consent	×	–	–	–
Demographic data	–	×	–	–
M.I.N.I.-KID	–	×	–	–
WISC	–	×	–	–
CANTAB	–	×	–	×
TMT	–	×	–	×
GazeFinder	–	×	–	×
SRS-2	–	×	–	–
SES	–	×	–	–
EHI	–	×	–	–
SSP	–	×	–	–
ACE	–	×	–	–
CTQ	–	×	–	–
JSQ-ES	–	×	–	–
JSQ-JH	–	×	–	–
WAI-SF	–	–	×	–
CSQ-8 (caregivers)	–	–	–	×
ADHD Rating Scale-5	–	×	–	×
Kid-KINDL questionnaire	–	×	–	×
Kiddo-KINDL questionnaire	–	×	–	×
SNABB	–	×	–	×
SDQ	–	×	–	×
DSRS-C	–	×	–	×
SCAS	–	×	–	×
Conners 3	–	×	–	×
BRIEF	–	×	–	×
DCDQ	–	×	–	–
CGI	–	×	–	×
PS (caregivers)	–	×	–	×
PSI-short form (caregivers)	–	×	–	×
PHQ-9 (caregivers)	–	×	–	×
GAD-7 (caregivers)	–	×	–	×
Brain MRI examination	–	×	–	×

M.I.N.I, Mini-International Neuropsychiatric Interview for Children and Adolescents; WISC, Wechsler Intelligence Scale for Children; CANTAB, Cambridge Neuropsychological Test Automated Battery; TMT, Trail Making Test; SRS-2, Social Responsiveness Scale, Second Edition; SES, Socio-economic status; EHI, Edinburgh Handedness Inventory; SSP, Short Sensory Profile; ACE, Adverse Childhood Experiences; CTQ, Childhood Trauma Questionnaire; WAI-SF, Working Alliance Inventory – Short Form; CSQ-8, Client Satisfaction Questionnaire–8; JSQ-ES, Japanese Sleep Questionnaire for Elementary Schoolers; JSQ-JH, Japanese Sleep Questionnaire for Junior High Schoolers; KINDL, Kinder Lebensqualität Fragebogen; SDQ, Strengths and Difficulties Questionnaire; DSRS-C, Depression Self-Rating Scale for Children; SCAS, Spence Children’s Anxiety Scale; BRIEF, Behavior Rating Inventory of Executive Function; DCDQ, Developmental Coordination Disorder Questionnaire; CGI, Clinical Global Impressions scale; PS, Parenting Scale; PSI, Parenting Stress Index; PHQ-9, Patient Health Questionnaire-9; GAD-7, Generalized Anxiety Disorder 7-item scale; MRI, magnetic resonance imaging; W, week.

### Study setting

This study will be conducted at the University of Fukui, and participant recruitment, screening, and informed consent procedures will be carried out at both the University of Fukui Hospital and the Hiratani Children’s Development Clinic. Participant recruitment commenced in March 2026, and the study will be conducted until April 2028. MRI data will be acquired at the University of Fukui Hospital using a PET/MR scanner following standardized imaging protocols to ensure data quality and consistency across participants.

### Recruitment of participants

Children with ADHD aged 6–15 years who express an interest in receiving ICBT will be prospectively recruited through outpatient clinical services at the University of Fukui Hospital and the Hiratani Children’s Development Clinic. Participants will first be assessed for eligibility in the multicenter ICBT clinical trial (ethics approval number: 2050220). Although this trial is conducted across multiple sites, participation in the present neuroimaging study will be limited to participants recruited from the two abovementioned sites. Among the participants who meet the eligibility criteria for the ICBT trial, those who provide additional consent and meet the inclusion and exclusion criteria for this neuroimaging study will be enrolled. ADHD will be diagnosed by qualified clinicians according to the Diagnostic and Statistical Manual of Mental Disorders, Fifth Edition, Text Revision (DSM-5-TR). Eligibility for the present study will be determined based on predefined inclusion and exclusion criteria (**Table 2**).

**Table 2:** The inclusion and exclusion criteria for children with attention-deficit/hyperactivity disorder.

Inclusion criteria	Exclusion criteria
1. Meets the DSM-5-TR diagnostic criteria for ADHD	1. A diagnosis of a severe organic brain disorder or PTSD
2. Aged between 6 and 15 years (inclusive) at the time of obtaining informed consent	2. An FSIQ < 80
	3. History of severe head trauma or neurological illness
	4. Any condition that may pose a physical or psychological risk during MRI examination, such as tattoos, implanted medical devices (e.g., pacemakers), pregnancy or possibility of pregnancy, claustrophobia, and fear of the dark

DSM-5-TR, Diagnostic and Statistical Manual of Mental Disorders, Fifth Edition, Text Revision; PTSD, post-traumatic stress disorder; FSIQ, full-scale intelligence quotient; ADHD, attention-deficit/hyperactivity disorder; MRI, magnetic resonance imaging.

TD children aged 6–15 years will be recruited from an ongoing neuroimaging database maintained by a research team at the University of Fukui (ethics approval number: 20220601) (26). At the time of protocol preparation, the ongoing database included 80 TD children aged 6–15 years (38 males [47.5%] and 42 females [52.5%]), with a mean age of 11.18 ± 2.60 years (range, 6.37–15.55 years). Because recruitment is ongoing, the final TD reference sample will be determined after conducting MRI quality control of all eligible participants available at the time of analysis. These TD children undergo MRI using the same scanner and the same imaging protocol as those used for the participants of this study. At enrollment, TD children will have no history of neurodevelopmental, psychiatric, or neurological disorders. MRI data from eligible TD participants will be used as the reference for both baseline neuroimaging comparisons and TD-like similarity analyses. The TD group will not undergo any intervention in this study.

### Medication management

Concomitant medications will be permitted throughout this study and recorded at each assessment. No medication washout will be required before MRI acquisition. Participants will be instructed to maintain their usual medication regimen during the study and not to initiate, discontinue, or modify medication use solely for study participation. Any medication changes during this study will be documented in the case report form and reported to the research team. These changes will be reviewed by the study investigators and considered in subsequent analyses. Participants with such changes will be retained in the primary analyses, and sensitivity analyses excluding these participants will be conducted to assess the robustness of the results.

### Randomization and blinding

Group assignments of the participants included in the neuroimaging study will be determined using the randomization procedure of the ICBT clinical trial. The present neuroimaging study includes a subset of participants from a randomized ICBT clinical trial and does not involve independent randomization. The MRI operators, outcome assessors, and investigators involved in MRI preprocessing and analysis will be blinded to the group allocation.

### Patient and public involvement

Patients and/or the public were not involved in the study design and will not be involved in the conduct, reporting, and dissemination of the findings of this study.

### Intervention

Both interventions will be delivered via the same secure web-based platform and will span an overall intervention period of 11 weeks, with asynchronous therapist support provided throughout the intervention period. The program will be completed jointly by the children and caregivers using age-appropriate materials. However, the interventions are not fully matched in treatment intensity or contact frequency. Participants in the ICBT group will receive a CBT-based program designed for children with ADHD and their caregivers, delivered weekly for 11 sessions (13). Participants in the sham control group will receive an online program without CBT-specific therapeutic components, consisting only of psychoeducation and parent training, delivered biweekly for a total of five sessions.

### Psychological measurements

Standardized psychological questionnaires will be used at baseline and post-intervention to assess the clinical symptoms and psychosocial functioning in children with ADHD, as well as caregiver-related outcomes. Parent-reported measures will be used to evaluate ADHD symptom severity, emotional and behavioral regulation, daily functioning, quality of life, and caregiver stress. The details of the questionnaires that will be used in this study are summarized in **Supplementary Table 1**. The validated Japanese versions of all questionnaires will be administered.

### Behavioral measurements

The Cambridge Neuropsychological Test Automated Battery (CANTAB; Cambridge Cognition, UK; https://cambridgecognition.com/) (27, 28) and the Trail Making Test (TMT) will be used to evaluate cognitive function in children with ADHD (29, 30). Response inhibition will be assessed using the Stop Signal Task from the CANTAB, in which the participants will be instructed to respond as quickly as possible to the direction of an arrow stimulus by selecting one of two response options and withholding their response when an auditory stop signal is presented. Spatial working memory will be evaluated using the Spatial Working Memory task of the CANTAB, which requires participants to search for hidden tokens within colored boxes on a screen and place them in a designated column. To minimize stereotyped search strategies, the participants will be instructed not to return to boxes in which a token has already been found. In addition, executive function and processing speed will be assessed using the TMT. Part A primarily measures visual scanning and processing speeds, whereas Part B assesses cognitive flexibility and set-shifting abilities.

The eye tracking system, GazeFinder (JVC Kenwood, Yokohama, Japan) (31), will be used to assess the visual attention and distractibility relevant to ADHD symptoms. Participants will view two sequentially presented video tasks designed to simulate attention-demanding classroom situations. Video 1 (30 seconds) will depict a simulated classroom lesson in which area of interest (AoI)-1 corresponds to the central instructional target (teacher and blackboard), and AoI-2 corresponds to peripheral distractors (e.g., clock, posters, and classmates). Video 2 (40 seconds) will present a competing stimuli paradigm in which AoI-1 displays a central target video, and AoI-2 includes peripheral distractors, with the distraction load gradually increasing. Eye gaze allocation to predefined AoIs, including fixation rates within the AoI-1 (target), AoI-2 (distractors), and non-AoI regions, as well as the frequency of gaze shifts between AoI-1 and AoI-2, will be recorded and analyzed.

### Imaging acquisition

For children with ADHD, MRI data will be acquired at two time points: before the start of the intervention (pre-intervention) and after the completion of the intervention (post-intervention). MRI will be performed at the University of Fukui Hospital using a 3.0-T GE Signa PET/MR scanner (General Electric HealthCare, Chicago, Illinois, USA), following standardized imaging protocols.

Before MRI acquisition, participants will undergo a standardized MRI familiarization procedure to improve compliance and reduce motion-related artifacts. Specifically, age-appropriate explanations of the MRI procedure will be provided using visual materials (e.g., an iPad); participants will listen to recorded scanner noise to familiarize themselves with the scanning environment, earplugs will be provided during image acquisition to reduce discomfort from scanner noise, and the participant’s head will be stabilized using foam padding to minimize head motion. These preparatory procedures are intended to reduce anxiety, improve cooperation, minimize head motion, and facilitate successful MRI acquisition. During rs-fMRI, participants will be instructed to think about nothing in particular, stay awake, and keep their eyes open and fixed on the crosshair on the screen. The imaging protocol will include rs-fMRI, T1w imaging, and DTI. The scanning parameters are listed in **Table 3**.

**Table 3:** Scanning parameters.

Protocol	Head coil	Scan time (min:s)	TR (ms)	TE (ms)	FA (°)	FOV (mm)	Matrix size	Voxel dimension (mm)	Slice thickness (mm)	Number of slices	Direction	b-values
	8 ch											
rs-fMRI		7:42	2300	30	81	192 × 192	64 × 64	3 × 3 × 3.5	3.5 (gap, 0.5)	40	N/A	N/A
T1w imaging		4:54	8.5	3.2	11	256 × 256	256 × 256	1 × 1 × 1	1.0 (gap, 0)	176	N/A	N/A
DTI		4:59	9352	74.6	N/A	240 × 240	128 × 128	1.9 × 1.9 × 3	3.0 (gap, 0)	45	30	0, 1000

rs-fMRI, resting-state functional magnetic resonance imaging; T1w, T1-weighted; DTI, diffusion tensor imaging; TR, repetition time; TE, echo time; FA, flip angle; FOV, field of view; N/A, not applicable; ch, channel.

### Primary outcome

The primary outcome is the intervention-related change in quantitative MRI-derived neural features, evaluated using the group × time interaction between group (ICBT vs. sham control) and time (pre- vs. post-intervention). These MRI features include indices of brain structure and function (e.g., regional brain volumes and cortical morphometry from structural MRI; FC and spontaneous neural activity measures, such as the amplitude of low-frequency fluctuations from rs-fMRI; and white matter microstructural indices, such as fractional anisotropy from DTI).

### Secondary outcomes

Secondary outcomes will be derived from the following analyses. 1) Baseline differences in MRI-derived neural markers between children with ADHD and TD children will be examined. 2) Between-group differences at the post-intervention time point will be examined to complement the primary analyses. 3) Associations between changes in MRI features and changes in clinical symptom measures will be explored within the ADHD group using standardized questionnaires and clinician-rated global impressions. 4) As an exploratory secondary outcome, the relationship between the baseline MRI features and treatment responses to ICBT will be examined to identify potential neural predictors of individual variability in treatment outcomes. 5) In addition, secondary outcomes will include cognitive performance measures obtained using the CANTAB and TMT, as well as eye-tracking indices obtained using the GazeFinder system.

### Data analysis plan

#### MRI data processing

MRI data collected before and after the intervention will be preprocessed and analyzed using standard neuroimaging software, such as SPM12 (https://www.nitrc.org/projects/spm/), CONN toolbox (32), DPABI (33), FreeSurfer (34), FSL (35), and MATLAB R2022a (MathWorks, Natick, MA, USA). Statistical analyses will be performed using SPSS (IBM SPSS Statistics, version 31; IBM Corp., Armonk, NY, USA) and R (https://www.r-project.org/).

The rs-fMRI data will be processed using the CONN toolbox and DPABI running in MATLAB. The CONN toolbox will be used for FC analyses, whereas DPABI will be used to calculate the amplitude of low-frequency fluctuations (ALFF) and regional homogeneity (ReHo). In both pipelines, the first 10 volumes will be discarded to allow signal stabilization. The remaining images will undergo slice-timing correction, realignment, and spatial normalization, followed by preprocessing and denoising procedures, including temporal band-pass filtering (0.01–0.08 Hz) and nuisance-signal regression. Nuisance regressors will include head-motion parameters and signals derived from white matter and cerebrospinal fluid. For FC analyses, spatial smoothing will be performed as part of the CONN preprocessing pipeline, and motion-related artifacts will be addressed using the default Artifact Detection Tools (ART)-based scrubbing procedure. For ALFF analyses, spatial smoothing will be performed before calculating the ALFF maps. For ReHo analyses, ReHo maps will be calculated from unsmoothed preprocessed data and subsequently spatially smoothed. Participants exhibiting head motion greater than 3.0 mm of translation and/or 3.0° of rotation will be excluded from further analyses. Mean framewise displacement (FD) will be calculated for each participant and included as a covariate in group-level resting-state fMRI analyses.

Structural MRI analyses will be performed using SPM12 and FreeSurfer software. CAT12 implemented in SPM12 will be used for voxel-based morphometric analyses of GMV, whereas FreeSurfer will be used for cortical and subcortical morphometric analyses, including cortical thickness and surface area, and subcortical volumes. Structural MRI quality will be assessed by visual inspection for motion artifacts, image distortion, and segmentation accuracy before statistical analyses.

DTI data will be analyzed using FSL. Standard preprocessing will include correction for image distortions and head motion before diffusion tensor estimation. Standard white matter indices, including fractional anisotropy (FA), mean diffusivity (MD), axial diffusivity (AD), and radial diffusivity (RD), will be calculated to evaluate the microstructural integrity of white matter. Whole-brain voxel-wise analyses of these diffusion metrics will subsequently be performed using tract-based spatial statistics (TBSS). No predefined region-of-interest or pathway-specific analyses are planned.

#### Primary analyses

Primary intervention effects will be examined using analysis methods appropriate to the format of each neuroimaging outcome. Whole-brain voxel-wise longitudinal analyses of GMV, ALFF, and ReHo maps will be performed using flexible factorial models implemented in SPM12, with subject, group (ICBT vs. sham control), and time (pre- vs. post-intervention) specified as factors. Subject will be included as a factor to account for repeated measurements within participants.

Longitudinal changes in DTI-derived diffusion metrics (FA, MD, AD, and RD) will be examined using whole-brain TBSS, with the group × time effect assessed as the between-group difference in pre- to post-intervention changes.

For MRI-derived quantitative measures represented as numerical values rather than whole-brain maps, including cortical thickness, surface area, subcortical volume, and ROI-to-ROI FC, linear mixed-effects models will be used. Group (ICBT vs. sham control), time (pre- vs. post-), and group × time interactions will be specified as fixed effects, with a participant-specific random intercept included to account for repeated measurements. As this study includes only two measurement occasions, random slopes will not be included. Model assumptions will be evaluated by visual inspection of residual and quantile–quantile plots in order to assess normality and homoscedasticity.

Between-group differences in intervention-related changes will be assessed primarily through the group × time interaction term, which will evaluate whether changes from pre- to post-intervention differ between the ICBT and sham control groups. Age and sex will be included as covariates in all primary analyses. If substantial between-group differences in handedness are observed, sensitivity analyses restricted to right-handed participants will be performed. Total intracranial volume will also be included in analyses of GMV and subcortical volume, whereas mean FD will be included in rs-fMRI analyses. When a significant group × time interaction is identified, follow-up simple-effects analyses will be performed to characterize the direction of the interaction, with appropriate adjustment for multiple comparisons. The main effects of group and time will also be reported as descriptive model terms.

#### Secondary analyses

As secondary analyses, cross-sectional comparisons will be performed to examine baseline neuroimaging differences between children with ADHD and TD children using pre-intervention ADHD and MRI data from an ongoing TD neuroimaging database acquired using the same MRI scanner and imaging protocol. General linear models adjusted for age, sex, and modality-specific covariates will be used for these comparisons. In addition, TD-like similarity analyses will be conducted to quantify the similarity between each participant’s individual neuroimaging pattern and the corresponding average neuroimaging pattern of the TD group. Pearson correlation coefficients will be calculated between the individual and TD reference patterns and subsequently Fisher z-transformed to derive individual similarity scores. Higher similarity scores will indicate a more TD-like neuroimaging pattern. Longitudinal changes in similarity scores and between-group differences in these changes will be examined using linear mixed-effects models.

#### Exploratory analyses

Exploratory analyses will examine whether baseline MRI markers are associated with individual differences in treatment response to ICBT. Treatment response will be defined as the change in the ADHD Rating Scale-5 (ADHD-RS-5) total score from pre- to post-intervention (ΔADHD-RS-5). Multiple linear regression analyses will be performed to determine whether baseline neuroimaging markers predict treatment response after adjusting for age, sex, and baseline ADHD-RS-5 total score. In addition, associations between neuroimaging measures and behavioral outcomes, including cognitive performance assessed using the CANTAB and TMT, as well as eye-tracking measures obtained using the GazeFinder system, will be explored using Pearson or Spearman correlation analyses, depending on the distribution of the variables. These analyses are considered hypothesis-generating and will be interpreted as exploratory.

#### Correction for multiple comparisons

For whole-brain voxel-wise analyses performed in SPM12, the statistical significance threshold will be set at a voxel-level threshold of p < 0.001 and family-wise error (FWE)-corrected cluster-level threshold of p < 0.05. For analyses involving multiple comparisons of ROI-based or numerical neuroimaging measures, the false discovery rate (FDR) will be controlled using the Benjamini–Hochberg procedure, with an FDR-adjusted p-value < 0.05 considered statistically significant. For TBSS analyses, statistical significance will be assessed using permutation-based nonparametric testing with threshold-free cluster enhancement (TFCE), with results considered significant at FWE-corrected p < 0.05.

#### Handling of missing data

All longitudinal MRI analyses will include only participants with evaluable MRI data at both pre- and post-intervention assessments. Analyses will be conducted using complete cases for the variables required in each analysis. Participants with missing values for variables required in a given analysis will be excluded from that analysis. The number of excluded cases and reasons for exclusion will be reported.

### Sample size

Based on feasibility considerations and sample sizes commonly reported in prior pediatric neuroimaging intervention studies (22, 36), the present study aims to recruit approximately 60 children with ADHD. Studies involving repeated MRI acquisitions combined with behavioral interventions in pediatric populations have typically included approximately 15–30 participants per group, reflecting practical constraints related to participant burden and data quality. Considering the expected attrition during the intervention period, as well as potential data loss due to MRI-specific factors (e.g., excessive head motion or incomplete scanning sessions), it is anticipated that approximately 40 participants (approximately 20 per intervention arm) will provide usable pre- and post-intervention MRI data and be included in the primary neuroimaging analyses. A formal power analysis was not performed because reliable effect-size estimates for longitudinal MRI studies evaluating ICBT in children with ADHD are currently unavailable.

### Ethics and dissemination

The study will be conducted in accordance with the Declaration of Helsinki. The study protocol was approved by the Research Ethics Committee of the University of Fukui Hospital in February 2026 (ethics approval number: 20250238). Written informed consent will be obtained from the parents or legal guardians, and assent will be obtained from the children. Participant recruitment commenced in March 2026 and is currently ongoing. The study findings will be disseminated through peer-reviewed publications and presentations at conferences.

## Discussion

This study aims to investigate the neural changes associated with an ICBT program in children with ADHD using multimodal MRI.

This study has several strengths. First, it leverages a randomized, sham-controlled ICBT clinical trial to examine intervention-related neural changes using longitudinal neuroimaging, an approach that is relatively rare in pediatric ADHD research. Although numerous neuroimaging studies have characterized brain function and structure in ADHD, most have employed a cross-sectional design. Importantly, studies examining changes in brain activity following CBT in children with ADHD are extremely limited, and existing intervention-related neuroimaging research has largely focused on pharmacological treatments (21, 37–40). By acquiring MRI data before and after ICBT, the present study enables the examination of within-individual neural changes associated with psychological interventions, providing novel insights into the potential neural mechanisms underlying CBT-based treatment effects.

Second, the inclusion of a sham control program allows for a more rigorous evaluation of intervention-specific neural changes. By comparing ICBT with a sham control condition delivered via the same web-based platform, this study attempts to minimize nonspecific effects, such as expectancy or engagement, which are particularly relevant in pediatric and digital intervention studies. This design strengthens the interpretability of the observed group-by-time interactions in MRI-derived markers.

Third, baseline neuroimaging data from TD children drawn from an ongoing neuroimaging database will provide an important reference for characterizing ADHD-related neural deviations at baseline. Although TD participants will not undergo longitudinal MRI as part of the present study, the cross-sectional comparisons may help contextualize whether intervention-related neural changes in children with ADHD shift toward the patterns observed in TD children. Such comparisons may contribute to a better understanding of the heterogeneity of ADHD-related brain function and structure.

In addition, this study explores associations between baseline neural markers and treatment response. Although the sample size limits definitive predictive modelling, examining such associations may offer preliminary insights into the potential neural characteristics related to differential responsiveness to ICBT. This exploratory approach is particularly relevant, given the growing interest in identifying neurobiological markers that may inform personalized interventions for neurodevelopmental disorders.

This study also has some limitations. First, the anticipated sample size, which is consistent with prior pediatric neuroimaging intervention studies, may limit the statistical power, particularly for voxel-wise analyses and exploration of associations. Accordingly, the findings should be interpreted cautiously. Second, the observed treatment-related neural changes and potential neuroimaging predictors will require further validation in larger, independent samples. Third, although a TD group will be included as a reference, only baseline MRI data from the TD group will be used in this study, whereas the ADHD group will undergo both pre- and post-intervention assessments. Therefore, changes observed after the intervention cannot be completely distinguished from those attributable to normal neurodevelopment. Fourth, although the sham control group was designed to control exposure to internet-based materials, it was not fully matched to the ICBT intervention in terms of treatment intensity and contact frequency (11 weekly sessions vs. 5 biweekly sessions). Therefore, between-group differences may partly reflect nonspecific factors, such as differences in participant engagement, treatment expectancy, or intervention exposure.

Despite these limitations, this study protocol provides a methodological framework for investigating the neural mechanisms of ICBT in children with ADHD using multimodal MRI. By prespecifying the study design, MRI acquisition procedures, and statistical analysis plan, the protocol intends to improve the transparency and reproducibility of future intervention-related neuroimaging research. Upon completion, this study is expected to provide new insights into brain changes associated with ICBT and address the limited evidence on its underlying neural mechanisms in children with ADHD. These findings may also inform future large-scale studies and facilitate the identification of objective neurobiological markers to guide individualized non-pharmacological treatment for children with ADHD.
