# Supplementary material for: Neural mechanisms of guided internet-based cognitive behavioral therapy for children with attention-deficit/hyperactivity disorder: a protocol for a prospective multimodal MRI study
Source: Front Psychiatry. 2026 Sep 23;17:1917984. doi: 10.3389/fpsyt.2026.1917984 (PMC13643404; doi:10.3389/fpsyt.2026.1917984)
Supplement: Supplementary file 1 [file Supplementaryfile1.docx]

Supplementary Material

# 1 Supplementary Table S1

| - **Table S1. Psychological measurements** | | |
| --- | --- | --- |
| - Questionnaire | - Item | - Assessment |
| - ADHD Rating Scale-5 ^1,2^ | 18-item; 4-point Likert scale (0 = never/rarely to 3 = very often) | - Severity of core ADHD symptoms (inattention and hyperactivity/impulsivity) |
| - SNABB ^3^ | Multiple items; 11-point numeric rating scale (0 = not at all concerning to 10 = extremely concerning) | - Parent-reported monitoring of ADHD symptoms over time |
| - SDQ ^4,5^ | 25-item; 3-point Likert scale (0 = not true to 2 = certainly true) | - Parent-reported emotional and behavioral difficulties and strengths in children |
| - DSRS-C ^6,7^ | - 18-item; 3-point Likert scale (0=not at all to 2=very) | - Depression |
| - SCAS ^8,9^ | - 38-item; 4-point Likert scale (0=never to 3=always) | - Symptoms of anxiety disorders |
| - Conners3 ^10^ | - 108-item (parents) and 99-item (children); 4-point Likert scale (0=not at all to 3=very) | - Inattentiveness and hyperactivity / impulsivity |
| - PSI-short form ^11,12^ | - 19-item; 5-point Likert scale (1=strongly disagree to 5=strongly agree) | Level of anxiety in interaction with their children and parental stress related to their children’s temperament and behavior |
| - PS ^13,14^ | - 30-item; 7-point Likert scale (1=effective discipline to 7=dysfunctional discipline) | - Discipline style in response to child misbehavior |
| - PHQ-9 ^15,16^ | 9-item; 4-point Likert scale (0 = not at all to 3 = nearly every day) | - Severity of depressive symptoms |
| - GAD-7 ^17,18^ | - 7-item; 4-point Likert scale (0 = not at all to 3 = nearly every day) | - Severity of anxiety symptoms |
| - WAI-short form ^19,20^ | - 12-item; 7-point Likert scale (1 = never to 7 = always) | - Perceived therapeutic alliance between caregivers and the gICBT program / therapists |
| - CSQ-8 ^21,22^ | 8-item; 4-point Likert scale (1 = quite dissatisfied to 4 = very satisfied) | - Satisfaction with the intervention program and perceived quality of care |
| - EHI ^23,24^ | - 10-item; typing a ‘+’ or ‘++’ in the appropriate column (right or left) | - Degree of hand laterality in daily activities |
| - SRS-2 ^25,26^ | - 65-item; 4-point Likert scale (1=not at all to 4=very) | - Social communication and restricted repetitive behavior |
| - Kid-KINDL^R^ questionnaire ^27,28^ | - 30-item; 5-point Likert scale (1=not at all to 5=very) | - Physical health, mental health, self-esteem, family, friends and school life |
| - Kiddo-KINDL^R^ questionnaire ^29,30^ | - 30-item; 5-point Likert scale (1=not at all to 5=very) | - Physical health, mental health, self-esteem, family, friends and school life |
| - JSQ-ES ^31^ | - 38-item; 6-point Likert scale (1=not at all to 6=very) | - Sleep disturbance and problematic sleep habits |
| JSQ-JH | - 38-item; 6-point Likert scale (1=not at all to 6=very) | - Sleep disturbance and problematic sleep habits |
| DCDQ^32,33^ | - 15-item; 5-point Likert scale (1=not at all like your child to 5=extremely like your child) | - Child’s gross and fine motor coordination |
| - SES ^34,35^ | - 5-item; single answer | - Parental education, occupation and monthly income |
| - BRIEF ^36,37^ | - 86-item; 3-point Likert scale (1=never to 3=often) | - Assessment of ability to inhibit, shift, control emotions, working memory and plan/organize |
| - ACE ^38,39^ | - 10-item; yes or no response | - Physical, verbal or sexual abuse, mental illness or substance abuse in the nuclear family |
| - CTQ ^40,41^ | - 28-item; 5-point Likert scale (1=never to 5=always) | - Childhood maltreatment and its severity |
| - CGI ^42^ | 2-item; 7-point clinician-rated scale | - Overall clinical severity and global improvement of symptoms |
| SSP ^43,44^ | - 38-item; 5-point Likert scale (1=not at all to 5=very) | - Hyperesthesia and insensitive |
| SDQ, Strengths and Difficulties Questionnaire; DSRS-C, Depression Self-Rating Scale for Children; SCAS, Spence Children’s Anxiety Scale; PSI, Parenting Stress Index; PS, Parenting Scale; PHQ-9, Patient Health Questionnaire-9; GAD-7, Generalized Anxiety Disorder 7-item scale; WAI-SF, Working Alliance Inventory – Short Form; CSQ-8, Client Satisfaction Questionnaire–8; EHI, Edinburgh Handedness Inventory; SRS-2, Social Responsiveness Scale, Second Edition; JSQ-ES, Japanese Sleep Questionnaire for Elementary Schoolers; JSQ-JH, Japanese Sleep Questionnaire for Junior High Schoolers; DCDQ, Developmental Coordination Disorder Questionnaire; SES, Socio-Economic Status; BRIEF, Behavior Rating Inventory of Executive Function; ACE, Adverse Childhood Experiences; CTQ, Childhood Trauma Questionnaire; CGI, Clinical Global Impressions scale; SSP, Short Sensory Profile. | | |

**Reference**

1.	DuPaul, G. J., Power, T. J., Anastopoulos, A. D. & Reid, R. *ADHD Rating Scale-5 for Children and Adolescents: Checklists, Norms, and Clinical Interpretation*. xi, 124 (The Guilford Press, New York, NY, US, 2016).

2.	Ishibashi, S. *et al.* Psychometrics of rating scales for externalizing disorders in Japanese outpatients: The ADHD‐Rating Scale‐5 and the Disruptive Behavior Disorders Rating Scale. *Int J Methods Psychiatr Res* **33**, e2015 (2024).

3.	Nylander, E., Hermansson, J. & Nygren, G. The SNABB ADHD treatment scale-An easy-to-use scale on treatment monitoring in childhood ADHD: A pilot study. *Front Child Adolesc Psychiatry* **2**, 1114565 (2023).

4.	Goodman, R. The Strengths and Difficulties Questionnaire: a research note. *J Child Psychol Psychiatry* **38**, 581–586 (1997).

5.	Tanabe, T. *et al.* Behavioral assessment of Japanese children with epilepsy using SDQ (strengths and difficulties questionnaire). *Brain Dev* **35**, 81–86 (2013).

6.	Birleson, P., Hudson, I., Buchanan, D. G. & Wolff, S. Clinical evaluation of a self-rating scale for depressive disorder in childhood (Depression Self-Rating Scale). *J Child Psychol Psychiatry* **28**, 43–60 (1987).

7.	Denda, K., Kako, Y., Kitagawa, N. & Koyama, T. Assessment of depressive symptoms in Japanese school children and adolescents using the Birleson Depression Self-Rating Scale. *Int J Psychiatry Med* **36**, 231–241 (2006).

8.	Spence, S. H. Structure of anxiety symptoms among children: a confirmatory factor-analytic study. *J Abnorm Psychol* **106**, 280–297 (1997).

9.	Ishikawa, S., Sato, H. & Sasagawa, S. Anxiety disorder symptoms in Japanese children and adolescents. *J Anxiety Disord* **23**, 104–111 (2009).

10.	Conners, C. K., Pitkanen, J. & Rzepa, S. R. Conners 3rd Edition (Conners 3; Conners 2008). in *Encyclopedia of Clinical Neuropsychology* 675–678 (Springer, New York, NY, 2008). doi:10.1007/978-0-387-79948-3_1534.

11.	Haskett, M. E., Ahern, L. S., Ward, C. S. & Allaire, J. C. Factor structure and validity of the parenting stress index-short form. *J Clin Child Adolesc Psychol* **35**, 302–312 (2006).

12.	Hatakeyama, T., Matsumura, K., Tsuchida, A., Inadera, H., & Japan Environment and Children’s Study (JECS) Group. Factor structure of the Parenting Stress Index-Short Form used in the Japan Environment and Children’s Study. *Sci Rep* **12**, 19123 (2022).

13.	Arnold, D. S., O’Leary, S. G., Wolff, L. S. & Acker, M. M. The Parenting Scale: A measure of dysfunctional parenting in discipline situations. *Psychological Assessment* **5**, 137–144 (1993).

14.	Itani, T. The Japanese version of the Parenting Scale: Factor structure and psychometric properties. *The Japanese journal of psychology* **81**, 446–452 (2010).

15.	Kroenke, K., Spitzer, R. L. & Williams, J. B. W. The PHQ-9. *J Gen Intern Med* **16**, 606–613 (2001).

16.	Inagaki, M. *et al.* Validity of the Patient Health Questionnaire (PHQ)-9 and PHQ-2 in general internal medicine primary care at a Japanese rural hospital: a cross-sectional study. *General Hospital Psychiatry* **35**, 592–597 (2013).

17.	Spitzer, R. L., Kroenke, K., Williams, J. B. W. & Löwe, B. A Brief Measure for Assessing Generalized Anxiety Disorder: The GAD-7. *Arch Intern Med* **166**, 1092–1097 (2006).

18.	Doi, S., Ito, M., Takebayashi, Y., Muramatsu, K. & Horikoshi, M. Factorial Validity and Invariance of the 7-Item Generalized Anxiety Disorder Scale (GAD-7) Among Populations With and Without Self-Reported Psychiatric Diagnostic Status. *Front Psychol* **9**, 1741 (2018).

19.	Munder, T., Wilmers, F., Leonhart, R., Linster, H. W. & Barth, J. Working Alliance Inventory-Short Revised (WAI-SR): psychometric properties in outpatients and inpatients. *Clinical Psychology & Psychotherapy* **17**, 231–239 (2010).

20.	Kawamura, A. *et al.* A Japanese Version of Working Alliance Inventory-Short Revised (J-WAI-SR): Development and Examination of Psychometric Properties. *Japanese Journal of Behavioral and Cognitive Therapies* **46**, 191–202 (2020).

21.	Attkisson, C. C. & Zwick, R. Client Satisfaction Questionnaire-8. https://doi.org/10.1037/t50119-000 (1982).

22.	Tatsumori, H. & Ito, H. Reliability and Validity of the Japanese Version of the 8-Item Client Satisfaction Questionnaire. *Seishin Igaku (Psychiatry)* **41**, (1999).

23.	Oldfield, R. C. The assessment and analysis of handedness: the Edinburgh inventory. *Neuropsychologia* **9**, 97–113 (1971).

24.	Hotta, C. Which inventory should be used to assess Japanese handedness? *Journal of Human Environmental Studies* **6**, 45–48 (2008).

25.	Kamio, Y. *et al.* Quantitative autistic traits ascertained in a national survey of 22 529 Japanese schoolchildren. *Acta Psychiatr Scand* **128**, 45–53 (2013).

26.	Constantino, J. N. Social Responsiveness Scale. in *Encyclopedia of Autism Spectrum Disorders* 4457–4467 (Springer, Cham, 2021). doi:10.1007/978-3-319-91280-6_296.

27.	Reiko, S. *et al.* A Study of the Kid-KINDL Questionnaire for Measuring Quality of Life in Elementary School Children in Japan. *Journal of the Japan Pediatric Society* **107**, 1514–1520 (2003).

28.	Helseth, S. & Lund, T. Assessing health-related quality of life in adolescents: some psychometric properties of the first Norwegian version of KINDL. *Scand J Caring Sci* **19**, 102–109 (2005).

29.	Kumiko, M. *et al.* A Study of the Kiddo-KINDL^R^ (Questionnaire for Measuring Health-Related Quality of Life in Children, Revised Version for 13 to 16-year-olds) in Japan. *Journal of the Japan Pediatric Society* **111**, 1404–1410 (2007).

30.	Yamaguchi, N. *et al.* Reliability and validity of a Nepalese version of the Kiddo-KINDL in adolescents. *Biosci Trends* **4**, 178–185 (2010).

31.	Kuwada, A. *et al.* Japanese Sleep Questionnaire for Elementary Schoolers (JSQ-ES): validation and population-based score distribution. *Sleep Med* **41**, 69–77 (2018).

32.	Nakai, A. *et al.* Evaluation of the Japanese version of the Developmental Coordination Disorder Questionnaire as a screening tool for clumsiness of Japanese children. *Res Dev Disabil* **32**, 1615–1622 (2011).

33.	Wilson, B. N. *et al.* Psychometric properties of the revised Developmental Coordination Disorder Questionnaire. *Phys Occup Ther Pediatr* **29**, 182–202 (2009).

34.	Rosenberg M. *Society and the adolescent self-image*. (Princeton University Press, 1965).

35.	Mimura, C. & Griffiths, P. A Japanese version of the Rosenberg Self-Esteem Scale: Translation and equivalence assessment. *Journal of Psychosomatic Research* **62**, 589–594 (2007).

36.	Gioia, G. A., Isquith, P. K., Guy, S. C. & Kenworthy, L. TEST REVIEW Behavior Rating Inventory of Executive Function. *Child Neuropsychology* **6**, 235–238 (2000).

37.	Momoda, M., Asano, R., Nagatani, F. & Taniike, M. Validation and normalization of the Japanese version of BRIEF among junior high school students. *The Japanese journal of psychology* **88**, 348–357 (2017).

38.	Felitti, V. J. *et al.* Adverse Childhood Experiences Study Questionnaire. https://doi.org/10.1037/t26957-000 (1998).

39.	Fujiwara, T. Impact of adverse childhood experience on physical and mental health: A life-course epidemiology perspective. *Psychiatry Clin Neurosci* **76**, 544–551 (2022).

40.	Bernstein, D. P., Ahluvalia, T., Pogge, D. & Handelsman, L. Validity of the Childhood Trauma Questionnaire in an Adolescent Psychiatric Population. *Journal of the American Academy of Child & Adolescent Psychiatry* **36**, 340–348 (1997).

41.	Mizuki, R. & Fujiwara, T. Validation of the Japanese version of the Childhood Trauma Questionnaire—Short Form (CTQ–J). *Psychological Trauma: Theory, Research, Practice, and Policy* **13**, 537–544 (2021).

42.	Busner, J. & Targum, S. D. The Clinical Global Impressions Scale. *Psychiatry (Edgmont)* **4**, 28–37 (2007).

43.	Iori, T. *et al.* Standardization of the Japanese Version of the Short Sensory Profile: Reliability and validity. *Seishin Igaku (Psychiatry)* **57**, 419–429 (2015).

44.	Mangeot, S. D. *et al.* Sensory modulation dysfunction in children with attention-deficit-hyperactivity disorder. *Dev Med Child Neurol* **43**, 399–406 (2001).

# 2 Supplementary Table S2

| **Table S2. List of abbreviations used in the manuscript** | |
| --- | --- |
| Abbreviation | Full term |
| ADHD | Attention-deficit/hyperactivity disorder |
| TD | Typically developing |
| CBT | Cognitive behavioral therapy |
| ICBT | Internet-based cognitive behavioral therapy |
| MRI | Magnetic resonance imaging |
| rs-fMRI | Resting-state functional magnetic resonance imaging |
| T1w | T1-weighted imaging |
| DTI | Diffusion tensor imaging |
| GMV | Gray matter volume |
| FC | Functional connectivity |
| ALFF | Amplitude of low-frequency fluctuations |
| ReHo | Regional homogeneity |
| FA | Fractional anisotropy |
| MD | Mean diffusivity |
| AD | Axial diffusivity |
| RD | Radial diffusivity |
| TIV | Total intracranial volume |
| FD | Framewise displacement |
| MATLAB | MATrix LABoratory |
| SPM12 | Statistical Parametric Mapping version 12 |
| CONN | CONN functional connectivity toolbox |
| DPABI | Data Processing & Analysis for Brain Imaging |
| FreeSurfer | FreeSurfer image analysis suite |
| FSL | FMRIB Software Library |
| SPSS | Statistical Package for the Social Sciences |
| CANTAB | Cambridge neuropsychological test automated battery |
| TMT | Trail making test |
| AoI | Area of interest |
| M.I.N.I.-KID | Mini-International Neuropsychiatric Interview for Children and Adolescents |
| WISC | Wechsler Intelligence Scale for Children |
| SRS-2 | Social Responsiveness Scale, Second Edition |
| SES | Socio-economic status |
| EHI | Edinburgh Handedness Inventory |
| SSP | Short Sensory Profile |
| ACE | Adverse Childhood Experiences |
| CTQ | Childhood Trauma Questionnaire |
| WAI-SF | Working Alliance Inventory – Short Form |
| CSQ-8 | Client Satisfaction Questionnaire–8 |
| ADHD-RS-5 | ADHD Rating Scale-5 |
| JSQ-ES | Japanese Sleep Questionnaire for Elementary Schoolers |
| JSQ-JH | Japanese Sleep Questionnaire for Junior High Schoolers |
| KINDL | Kinder Lebensqualität Fragebogen |
| SDQ | Strengths and Difficulties Questionnaire |
| DSRS-C | Depression Self-Rating Scale for Children |
| SCAS | Spence Children’s Anxiety Scale |
| BRIEF | Behavior Rating Inventory of Executive Function |
| DCDQ | Developmental Coordination Disorder Questionnaire |
| CGI | Clinical Global Impressions scale |
| PS | Parenting Scale |
| PSI | Parenting Stress Index |
| PHQ-9 | Patient Health Questionnaire-9 |
| GAD-7 | Generalized Anxiety Disorder 7-item scale |
| DSM-5-TR | Diagnostic and Statistical Manual of Mental Disorders, Fifth Edition, Text Revision |
| PTSD | Post-traumatic stress disorder |
| FSIQ | A full-scale intelligence quotient |
| TR | Repetition time |
| TE | Echo time |
| FA | Flip angle |
| FOV | Field of view |
| N/A | Not applicable |
| ch | channel |
